# A Comprehensive Free-Breathing Protocol for Cardiovascular Magnetic Resonance Imaging of Ischemia and Cardiomyopathies: a Feasibility Study

**DOI:** 10.1186/1532-429X-18-S1-P313

**Published:** 2016-01-27

**Authors:** Juliano L Fernandes, Luciana A Fioravante, Michael O Zenge, Christoph Forman, Michaela Schmidt, Mariappan S Nadar, Paulo E Mazo, Andreas Greiser, Peter Speier, Daniel Staeb, Hui Xue, Michael S Hansen, Peter Kellman, Ralph Strecker

**Affiliations:** 1 Cardiovascular Imaging, Jose Michel Kalaf Research Institute, Campinas, Brazil; 2 Siemens Healthcare Diagnosticos SA, Sao Paulo, Brazil; 3 grid.94365.3d0000000122975165National Heart, Lung and Blood Institute, National Institutes of Health, Bethesda, MD USA; 4 Siemens Healthcare GmbH, Erlangen, Germany; 5 Siemens Medical Solutions USA Inc, Princeton, NJ USA; 6 grid.8379.50000000119588658Diagnostic and Interventional Radiology, University of Wuerzburg, Wuerzburg, Germany; 7 grid.1003.20000000093207537Centre for Advanced Imaging, University of Queensland, Brisbane, QLD Australia

## Background

Despite recent advances in cardiovascular magnetic resonance imaging, exams are still considered long and challenging from a technical perspective as well as demanding for patients who have to repeatedly perform multiple breath-holds (BH). Free-breathing (FB) sequences have been developed for distinct applications but they are usually performed in isolation within a traditional protocol that still requires BH based acquisitions. We studied the feasibility of a complete FB exam for the assessment of ischemia and cardiomyopathies integrating different prototype sequences.

## Methods

Six sequential patients underwent the FB protocol and were retrospectively compared to twelve patients matched for sex, age and type of study who had undergone a routine exam at 1.5T (Magnetom Siemens Aera). The FB protocol comprised orthogonal and cardiac axis localizers, axial black-blood T1w half Fourier single-shot turbo spin echo (HASTE) images, a prototype FLASH perfusion sequence with simultaneous multi-slice acquisition, a prototype cine SSFP sequence with sparse sampling and iterative reconstruction (SSIR) and a prototype respiratory motion-corrected SSFP averaged phase-sensitive inversion recovery images (MOCO-PSIR-LGE). MOCO-PSIR-LGE images were reconstructed rapidly in-line using the Gadgetron framework [1]. Spatial and temporal resolution of the FB sequences matched the traditional protocol which acquired all images during BH including localizers, HASTE axial image FLASH perfusion images, SSFP cines, and 2D single-shot acquisition with SSFP readout for LGE. The two protocols were compared for exam time and overall image quality using the average of the sum of each individual component (perfusion, cine and LGE) on a 4-point scale (3=excellent, 2=good with minor artifacts, 1=diagnostic but with major artifacts, 0=non diagnostic).

## Results

**Figure 1:**
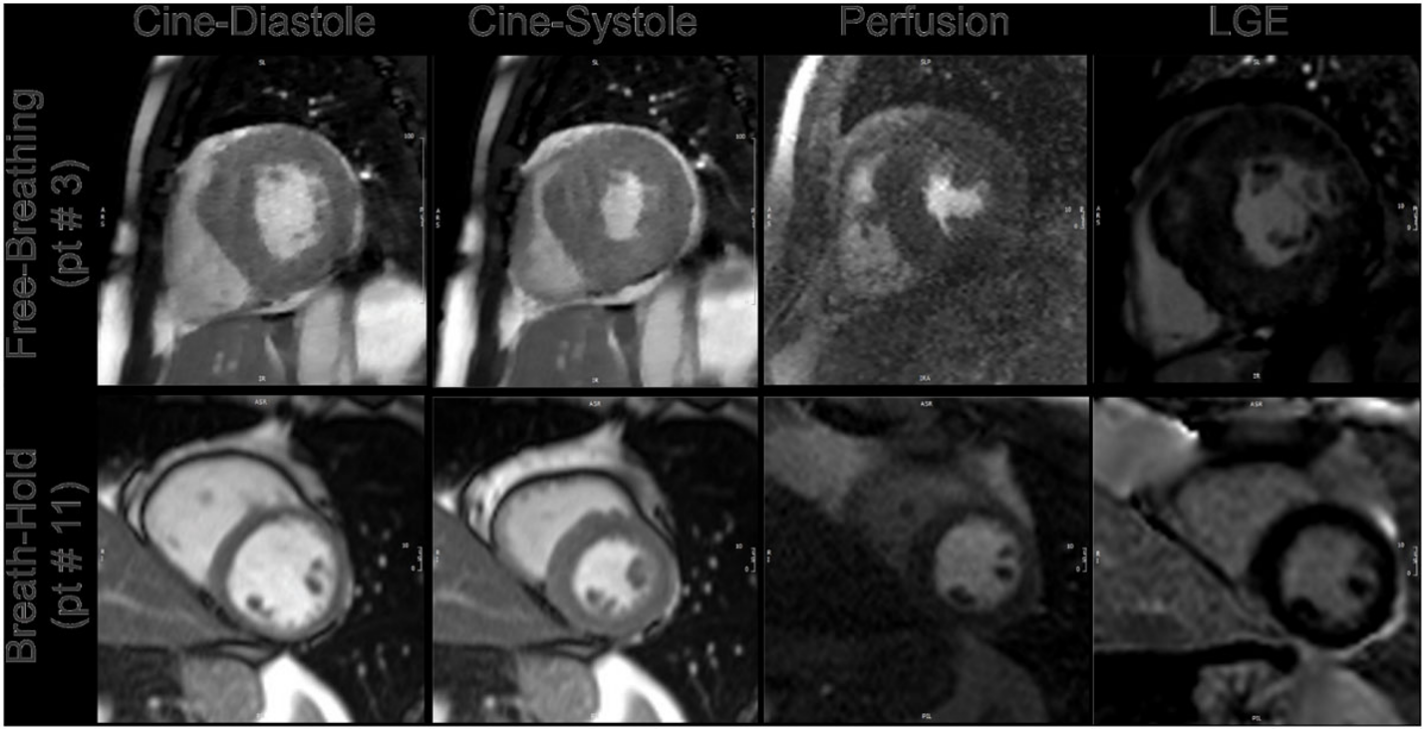
Figure 1

There were no baseline differences between the two groups (average age of 57.6 ± 14.2 years; 67% males). All patients completed the FB protocol (two for ischemia, four for cardiomyopathy assessment). The mean time for the FB protocol was 21.0 ± 5.1 minutes (range 14 to 27 minutes), significantly shorter than patients that underwent the traditional BH protocol with 27.5 ± 5.3 minutes (range 19 to 37 minutes), P = 0.02. Image quality analysis did not show any significant differences comparing the two protocols with an overall score for the FB images of 2.6 ± 0.25 versus 2.7 ± 0.24 for the traditional protocol (P = 0.37) with samples images shown in Figure 1. The quality score for each individual component of the exam did not show any differences.

## Conclusions

A complete FB protocol integrating new prototype sequences is feasible and allows for faster imaging times while maintaining the overall quality of cine, perfusion and LGE images. This FB protocol may not only simplify the exam and make it faster but also improve the robustness of the method in difficult patients where traditional BH sequences perform poorly.
